# Supplementary material for: Venomix: a simple bioinformatic pipeline for identifying and characterizing toxin gene candidates from transcriptomic data
Source: PeerJ. 2018 Jul 31;6:e5361. doi: 10.7717/peerj.5361 (PMC6074769; doi:10.7717/peerj.5361)
Supplement: Supplemental Information 3 [file peerj-06-5361-s003.gz › FinalOutput_E-20/Zinc_metalloproteinase@disintegrin_107/finaltree.pdf]

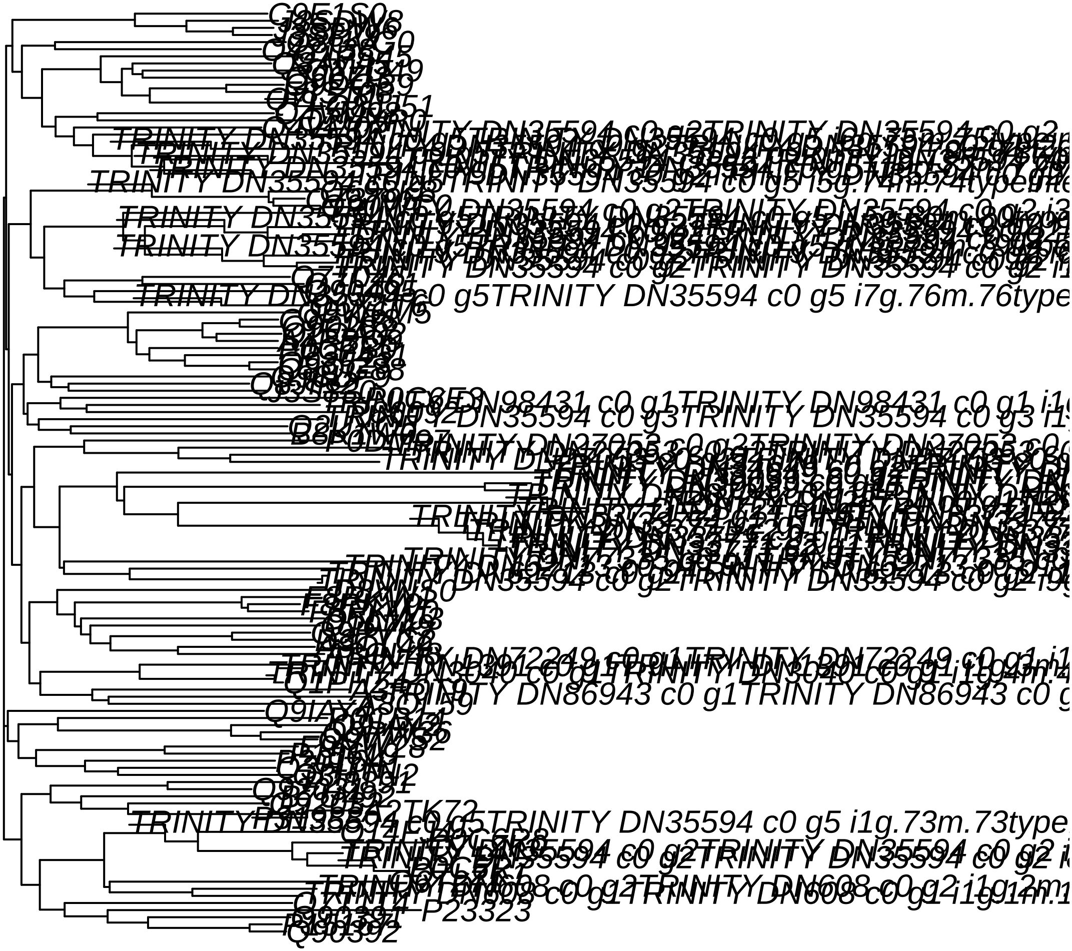
G9F1S0
TRINITY DN35594 c0 g5TRINITY DN35594 c0 g5 i7g.76m.76type
TRINITY DN98431 c0 g1TRINITY DN98431 c0 g1 i1g
TRINITY DN72249 c0 g1TRINITY DN72249 c0 g1 i1
TRINITY DN86943 c0 g1TRINITY DN86943 c0 g
TRINITY DN35594 c0 g5TRINITY DN35594 c0 g5 i1g.73m.73type
TRINITY DN608 c0 g1TRINITY DN608 c0 g1 i1g1m.1
P23323
Q90392
